# Hepatitis C Virus Elimination Program among Prison Inmates, Israel

**DOI:** 10.3201/eid2911.230728

**Published:** 2023-11

**Authors:** Lihi Eisen, Zohar Mor, Miriam Madar, Ron Rabinovitch, Yuval Dadon, Rivka Sheffer, Ehud Kaliner, Liav Goldstein

**Affiliations:** Ministry of Health, Tel-Aviv Department of Health, Tel Aviv, Israel (L. Eisen, R. Sheffer);; Ashkelon Academic College, Ashkelon, Israel (Z. Mor);; Central Department of Health, Ministry of Health, Ramla, Israel (Z. Mor, E. Kaliner);; Israel Prison Service, Israel (M. Madar, R. Rabinovitch, L. Goldstein);; Deputy Director Office, Ministry of Health, Jerusalem, Israel (Y. Dadon)

**Keywords:** Hepatitis C, hepatitis C virus, HCV, viruses, correctional facilities, drug users, prisoners, screening, treatment, Israel

## Abstract

The Israeli Prison Services implemented a hepatitis C virus (HCV) elimination program in 2020. Inmates considered high risk for HCV were offered serology; HCV-seropositive participants were offered HCV RNA testing. Among participants, 7.0% had detectable HCV RNA and were offered antiviral drug therapy. This program reduced HCV burden among incarcerated persons.

In 2019, the Israeli Ministry of Health joined the World Health Organization initiative to eliminate hepatitis C virus (HCV) (*1*). The national elimination program included HCV testing among 6 high-risk groups: recipients of blood transfusions before 1992; persons who use intravenous drugs; patients treated with improperly sterilization medical equipment or blood products; immigrants from the former Soviet Union and Romania; persons infected with HIV or hepatitis B virus (HBV); and known HCV carriers (*2*).

HCV seroprevalence among the general population of Israel is estimated to be 1.9% and detectable HCV RNA is estimated to be 0.7% (*3*,*4*). In Israel, the prevalence of HCV RNA among migrants from the former Soviet Union is higher than that of the general population, reaching ≈5.5% (*5*); seroprevalence is ≈60.0% among intravenous drug users (IVDUs) (*6*).

In August 2020, the Israeli Prison Service (IPS), which operates 33 prisons and houses ≈14,500 prisoners across the country (*7*), implemented an HCV screening, testing, and treatment initiative among inmates at high risk for HCV. We estimated HCV prevalence among prison inmates and identified demographic and behavioral characteristics of HCV-infected inmates.

## The Study

HCV serology screening tests were offered to all inmates considered to be at high risk for HCV infection if they met >1 of the following criteria: had a history of illicit drug use or were arrested for a drug-related felony; were born in the former Soviet Union or Romania; or had known HIV or HBV infection. Inmates were screened and followed from August 1, 2020, through July 31, 2021, or prison discharge. We collected data from IPS medical records and only included inmates incarcerated for >30 days to allow sufficient time to complete the screening process.

Willing inmates who were at high risk for HCV were screened for HCV antibodies and, if positive, were screened for HCV RNA. Participants with detectable HCV RNA were offered free, direct-acting antiviral drug treatment after consultation with gastroenterologists. We considered undetectable HCV RNA 12 weeks after treatment to be sustained viral response. Injection drug use is prohibited in prison; thus, we defined IVDUs as inmates who were treated with opiate substitutes.

We compared HCV-seropositive and seronegative participants. We used the Student *t*-test to compare continuous variables and the χ*^2^* test for categorical variables. We considered p<0.05 statistically significant. We performed multivariate analysis by logistic regression to identify variables associated with being HCV seropositive and calculated odds ratios and 95% CIs. The study was approved by the ethics committee of Ashkelon Academic College in Israel (approval no. 2022–33).

Serologic screening was offered to all 2,824 inmates in the IPS who were at high risk for HCV infection; 1,751 (62.0%) opted to be tested and 1,021 (36.2%) declined (Figure). Of the tested inmates, 21.6% (379) were seropositive. HCV RNA testing was performed in 317 (83.6%) of the positive serology group and 119 were positive; 47 declined testing, and 15 were released from prison before testing. Therefore, detectable HCV RNA was found in 7.0% (119/1,689) of all tested participants. Of those, 113 had newly diagnosed cases and 6 had known cases.

**Figure:**
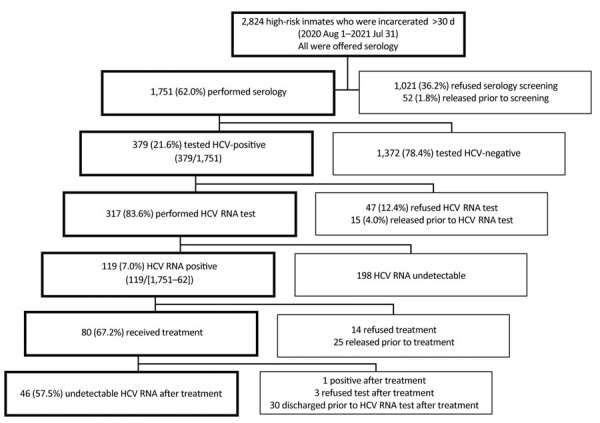
Flowchart of participant enrollment in a hepatitis C elimination program among high-risk prison inmates, Israel. Bold boxes on left indicate participants. HCV, hepatitis C virus.

Altogether, 80/119 (67.2%) inmates completed antiviral drug treatment during incarceration. Of those followed up after 12 weeks, 97.8% (46/47) had confirmed sustained viral response; the other 33 refused follow-up testing or were discharged from prison.

Compared with nonparticipants, the participant group comprised more female inmates, married persons, nonsmokers, IVDUs, and HIV- or HBV-infected persons; participants were also older and had fewer previous incarcerations (Table 1). Inmates who were HCV seropositive were more likely older, single, had been previously incarcerated, were born in the former Soviet Union or Romania, had used intravenous drugs, or were HIV- or HBV-positive (Table 2). In the multivariate analysis, older age, being single, having a greater number of previous incarcerations, being born in the former Soviet Union or Romania, drug use, and HBV infection were variables predicting HCV-positive serology among participants.

**Table 1:** Characteristics of participants and nonparticipants in a hepatitis C virus elimination program among high-risk prison inmates, Israel*

Characteristics	Participated in serology screening, n = 1,751	Did not participate in serology screening, n = 1,021	p value
Gender			0.01
M	1,701 (97.1)	1,009 (98.8)	
F	49 (2.8)	12 (1.2)	
Transgender	1 (<0.1)	0	
Mean age, y (SD)	41.6 (12.2)	36.4 (11.1)	<0.01
Marital status			
Married	454 (25.9)	193 (18.9)	<0.01
Nonmarried†	1,282 (73.2)	812 (79.5)	
Data not available	15 (0.9)	16 (1.6)	
Education level, did not complete high school	1,358 (77.5)	810 (79.3)	0.2
Previously incarcerated	1,471 (84.0)	922 (90.3)	<0.01
Mean number of imprisonments (SD)	8.2 (7.3)	8.7 (6.7)	<0.01
Smoker	1,442 (82.3)	885 (86.6)	0.03
Alcohol user	202 (11.5)	99 (9.7)	0.1
Risk criteria			
Country of birth			
Former Soviet Union and Romania	651 (37.2)	288 (28.2)	<0.01
Israel	876 (50.0)	599 (58.7)	
West Bank and Gaza Strip	115 (6.6)	54 (5.3)	
Other countries	80 (4.6)	62 (6.1)	
Data not available	29 (1.6)	18 (1.7)	
Drug use			
No drug use	371 (21.2)	182 (17.9)	0.03
Non–IV drug use	1,053 (60.1)	718 (70.3)	<0.01
IV drug use	327 (18.7)	121 (11.8)	<0.01
HIV infection	18 (1.0)	2 (0.1)	0.01
Hepatitis B virus infection	71 (4.0)	13 (0.7)	<0.01

*Values indicate no. (%) unless otherwise indicated. IV, intravenous. †Includes single, divorced, and widowed.

**Table 2:** Comparison between participants with HCV-positive and HCV-negative serology in a hepatitis C virus elimination program among high-risk prison inmates, Israel*

Characteristics	HCV-positive, n = 379	HCV-negative, n = 1,372	p value	Multivariate analysis	
				OR (95% CI)	p value
Sex					
M	367 (96.8)	1,334 (97.2)	0.8		
F	12 (3.2)	37 (2.7)			
Transgender	0	1 (0.1)			
Mean age, y (SD)	47.6 (9.3)	39.9 (12.4)	<0.01	1.06 (1.05–1.08)	<0.01
Marital status					
Married	77 (20.3)	375 (27.33)	<0.01	Referent	<0.01
Nonmarried†	299 (78.9)	983 (71.65)		1.7 (1.2–2.4)	
Data not available	3 (0.8)	14 (1.02)			
Education level, did not complete high school	299 (78.9)	1,059 (77.2)	0.4		
Previously incarcerated‡	342 (90.2)	1,129 (82.2)	<0.01		
Mean number of imprisonments (SD)	11.6 (8.8)	7.3 ± 6.5	<0.01	1.07 (1.05–1.09)	<0.01
Smoker	324 (85.5)	1,118 (81.5)	0.07		
Alcohol user	33 (8.7)	169 (12.3)	0.05		
Risk criteria					
Country of birth					
Former Soviet Union and Romania	201 (53.0)	450 (32.8)	<0.01	7.4 (5.4–10.3)	
Israel	157 (41.4)	719 (52.4)		Referent	<0.01
West Bank and Gaza Strip	9 (2.4)	106 (7.7)			
Other countries	9 (2.4)	71 (5.2)			
Data not available	3 (0.8)	26 (1.9)			
Drug use					
No drug use	53 (14.0)	318 (23.2)	<0.01	Referent	<0.01
Non–IV drug use	176 (46.4)	877 (63.9)		5.4 (3.5–8.4)	
IV drug use	150 (39.6)	177 (12.9)		12.8 (7.9–20.7)	
HIV infection	11 (2.9)	7 (0.5)	<0.01	NA	NS
Hepatitis B virus infection	31 (8.1)	40 (2.9)	<0.01	3.8 (2.0–7.3)	<0.01

*Values indicate no. (%) unless otherwise indicated. IV, intravenous; NA, not applicable; NS, not significant. †Includes single, divorced, and widowed. ‡This variable was not included in the multivariate analysis because it is collinear with number of imprisonments.

## Conclusions

HCV seroprevalence among study participants was 21.6% and detectable HCV RNA was 7.0%. Those prevalences are higher than the estimates of 1.9% seroprevalence and 0.7% HCV RNA-positive in the general population of Israel (*3*,*4*). However, HCV seroprevalence among study participants was lower than persons in the general population who use intravenous drugs (60.0%) (*6*), and close to HCV RNA prevalence (≈5.5%) among male migrants from former Soviet Union in the general population (*5*).

The rates of HCV infection among inmates vary widely in different prison populations worldwide. A previous meta-analysis (*8*) explained HCV seroprevalence heterogeneity (2.0%–58.0%) in the correctional system mainly by differences in proportion of inmates who are IVDU (3.0%–69.0%). In our study, seroprevalence was 21.6% and the proportion of IVDUs among inmates who participated in the study was 18.6%.

Older age, being single, having a greater number of previous incarcerations, being born in the former Soviet Union or Romania, and being HBV-positive were risk factors associated with HCV-positive serology among participants in our study. Inmates who had used intravenous drugs had higher rates of HCV-positive serology, as previously published (*9*). Of note, prisoners who used drugs by other than intravenous routes and those who were incarcerated for drug-related felonies also had higher rates of HCV-positive serology. Because most risk factors can be identified by prison authorities when inmates are admitted to prison, IPS can use a targeted screening policy to improve HCV elimination.

A total of 67.2% of infected inmates completed antiviral drug treatment during their sentences. That completion rate helped lower the overall HCV burden in IPS and highlights the contribution of the screening program to decrease HCV in prison settings among persons at high-risk for HCV. The IPS plays a critical role in the national efforts to achieve HCV elimination in Israel. In line with the high turnover of inmates who revolve between the community and prisons, the success in reducing HCV burden during incarceration also positively affects the community (*10*). IPS structural efforts, inmate cooperation (*11*), and involvement of the medical insurers in the community are all essential to accomplishing HCV elimination in Israel.

The first limitation of this study is that the data were collected from IPS medical files, and we had limited access to community medical records of inmates who were discharged from prison. Thus, antiviral drug treatment outcomes in those cases could not be determined. Second, not all eligible inmates were willing to be screened, which might lead to selection bias. To assess the bias, we compared the characteristics of those refusing to those who were tested and found lower proportions of all individual high-risk criteria among those who declined screening (Table 1). Thus, the bias is conservative if it exists. Third, acute cases of HCV might not have been detected in serologic testing due to a window period. To increase internal validity, only inmates whose incarceration was >1 month were included. Fourth, some of the data, such as smoking history, could be subject to reporting bias. Last, this study did not classify HCV clusters (*12*) because of lack of available data.

In conclusion, HCV seroprevalence among inmates at high risk was 21.6% and HCV RNA was detectable in 7.0%. The risk factors associated with HCV-positive serology among inmates included older age, being single, and having more previous incarcerations, as well as other HCV risk criteria, such as IV drug use, being born in former Soviet Union or Romania, and being co-infected with HBV. The IPS-led HCV elimination program achieved a decrease in HCV burden among inmates at high risk for HCV. To achieve national HCV elimination, Israel should strengthen cooperation between IPS and medical insurers in the community.
